# Array-Based Screening of Silver Nanoparticle Mineralization Peptides

**DOI:** 10.3390/ijms21072377

**Published:** 2020-03-30

**Authors:** Masayoshi Tanaka, Shogo Saito, Reo Kita, Jaehee Jang, Yonghyun Choi, Jonghoon Choi, Mina Okochi

**Affiliations:** 1 Department of Chemical Science and Engineering, Tokyo Institute of Technology, 2-12-1, O-okayama, Meguro-ku, Tokyo 152-8552, Japan; tanaka.m.bn@m.titech.ac.jp (M.T.); saito.s.bc@m.titech.ac.jp (S.S.); 2 School of Science; Tokyo Institute of Technology, 2-12-1, O-okayama, Meguro-ku, Tokyo 152-8552, Japan; kita@stat.phys.titech.ac.jp; 3 School of Integrative Engineering, Chung-Ang University, Seoul 06974, Korea; jjaeh95@gmail.com (J.J.); dydgus5057@gmail.com (Y.C.); jonghoonc@gmail.com (J.C.)

**Keywords:** AgNP, peptide array, biomineralization, green synthesis

## Abstract

The use of biomolecules in nanomaterial synthesis has received increasing attention, because they can function as a medium to produce inorganic materials in ambient conditions. Short peptides are putative ligands that interact with metallic surfaces, as they have the potential to control the synthesis of nanoscale materials. Silver nanoparticle (AgNP) mineralization using peptides has been investigated; however, further comprehensive analysis must be carried out, because the design of peptide mediated-AgNP properties is still highly challenging. Herein, we employed an array comprising 200 spot synthesis-based peptides, which were previously isolated as gold nanoparticle (AuNP)-binding and/or mineralization peptides, and the AgNP mineralization activity of each peptide was broadly evaluated. Among 10 peptides showing the highest AgNP-synthesis activity (TOP10), nine showed the presence of EE and E[X]E (E: glutamic acid, and X: any amino acid), whereas none of these motifs were found in the WORST25 (25 peptides showing the lowest AgNP synthesis activity) peptides. The size and morphology of the particles synthesized by TOP3 peptides were dependent on their sequences. These results suggested not only that array-based techniques are effective for the peptide screening of AgNP mineralization, but also that AgNP mineralization regulated by peptides has the potential for the synthesis of AgNPs, with controlled morphology in environmentally friendly conditions.

## 1. Introduction

Different metallic nanoparticles, such as silver nanoparticles (AgNPs), have received marked attention in various fields including molecular labeling [1], sensing [2], microbiocidal activities [3], and catalysis [4]. To expand the characteristics of these molecules, such as optical, electronic, and catalytic properties, numerous studies have focused on the regulation of AgNP synthesis. However, the controlled synthesis of AgNPs, especially in an aqueous solution through a green synthesis course, is still challenging. Hence, it is necessary to develop a technique to synthesize AgNPs, controlling their morphology, size, and properties [5,6].

Biological molecules directed at the synthesis of metallic nanoparticles have received great attention in recent years, due to their potential as green and economic synthesis methods [7,8,9]. Whereas various biological molecules, including pigments, nucleic acids and proteins are utilized for nanoparticle synthesis and functionalization [7,8,9,10,11,12,13], peptides also comprise promising ligand molecules that bind, not only metallic ions, but also metallic crystals. This is because variants are designed abundantly from combinations using amino acids with various physicochemical properties through chemical synthesis [14,15,16,17,18]. Among a wide range of functional peptides, catalytic peptides used for nanoparticle synthesis regulation, named mineralization peptides, have commonly been isolated from peptides that are strongly bound to target crystals [14,15,16]. Considering the chemical equilibrium of crystalline nanoparticles and their metallic ions in solution, it has been suggested that the strong binding of peptides stabilizes the crystals, resulting in a shift in the equilibrium from an ionic state towards that of the crystal; specifically, target crystal mineralization is mediated by peptide addition. AgNP mineralization by peptides has been investigated for the development of an AgNP-synthesis technique in ambient conditions [19,20]; however, detailed analyses of peptide directed-AgNP property design are still required. 

In this research area, one of the limitations is the number of known AgNP mineralization peptides. To evaluate the mineralization mechanism, additional AgNP-mineralization peptides are required. Among the various techniques used for peptide screening (e.g., phage display library, cell surface display library, ribosome library), coherent membrane-supported peptide array libraries based on spot-synthesis are known to have different advantages, such as ease of peptide sequence identification (DNA sequencing is not necessary), chemical synthesis without a biological organism, and the gain of mineralization activity data from positive (high-mineralization peptides) to negative (weak- or null-mineralization) with amino acid sequences [21,22]. In a previous study, we developed a technique to screen nanoparticle-binding peptides using the peptide array technique [23,24,25,26]. Among them, gold nanoparticle (AuNP)-binding peptides were isolated through the design of an array based on variations in the amino acid frequency, informed by empirical results of their binding assays [25]. This simple strategy resulted in approximately 1800 peptides with various AuNP-binding affinities. 

In this study, we explored the green synthesis of AgNPs, using peptides without any environmentally hostile chemicals (e.g., NaBH_4_). To investigate the AgNP mineralization by peptides, peptide array technology was used to identify a list of mineralization peptides with various physicochemical properties. The peptide library was designed from an AuNP-binding peptide, previously reported, because many of these peptide sequences revealed AuNP mineralization activity [15,25]. To expand the potential for other nanoparticle synthesis, the top 200 AuNP-binding peptides were herein evaluated in terms of AgNP mineralization activity. 

## 2. Results

### 2.1. Screening of AgNP Mineralization Peptide Using Peptide Array Consisting of AuNP-Binding Peptides

**Figure 1:**
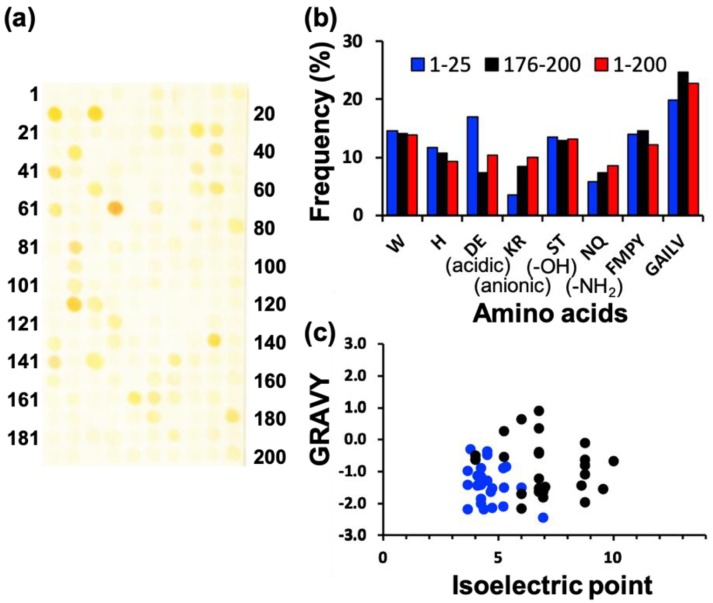
Screening of silver nanoparticle (AgNP) mineralization peptides using peptide array consisting of AuNP-binding peptides. (**a**) Representative image of peptide array after biomineralization reaction by soaking in AgNO_3_-containing solution for 7 h. (**b**) Amino acid frequencies for high-mineralization peptides (TOP25) and low-mineralization peptides (WORST25), based on the brightness evaluation from each peptide spot using ImageJ. (**c**) Physicochemical properties of high- and low-mineralization peptides. Physicochemical properties of high-binding TOP25 peptides (blue circle) and worse binding WORST25 peptides (black circle), based on pI and GRAVY (the grand average of hydropathy) values. The latter is considered the average hydropathy value for all amino acids in a peptide sequence; therefore, high GRAVY values denote hydrophobicity.

To screen various types of AgNP mineralization peptides, AgNP mineralization properties of the top 200 (TOP200) high AuNP-binding peptide sequences (Table S1 in [15]) were investigated. Based on the result shown in Figure 1a, approximately 50 AgNP mineralization peptides were isolated using the peptide array. After a 7-h incubation of the peptide array in an aqueous solution of 50 mM AgNO_3_ in MilliQ water, individual peptide array spots were observed to change color, indicative of AgNP mineralization. The mineralization profiles using a peptide array with the same peptide library were not identical to the results of an AuNP mineralization evaluation previously reported [15]. From this observation, the mineralization mechanism associated with peptides seems to be different between AuNP and AgNP mineralization. The observed colors were yellowish and consistent at all mineralizing peptide spots, except those of peptide 64 (AESEHEWEVA) and 112 (NWELEEHSAS) (Figure 1a), showing an orange-yellow color. 

In addition, comparative analyses of amino acid frequencies and sequences among the TOP25 peptides are shown in Figure 1b. Comparing the amino acid frequency with the average of all 200 library peptides, results indicated a significantly higher proportion of acidic amino acids, including aspartic acid (D) and glutamic acid (E) monomers in AgNP mineralization peptides, whereas these were notably reduced in the bottom 25 (WORST25) peptides. It is probable that this results from the negative charge of amino acids, which can interact with Ag^+^. In terms of other minerals of magnetite and hydroxyapatites, the importance of these amino acids has also been reported; however, the mechanisms are still controversial [22,23,24]. Therefore, these residues could comprise a prerequisite for all AgNP biomineralization peptides. The importance of acidic amino acids was also supported by the plot analysis of peptide physicochemical properties (Figure 1c). Almost all peptides were found in a region of hydrophilicity and a low isoelectric point (pI), whereas the bottom 25 (WORST25) peptides were widely spread in the chart. The amino acid frequency and physicochemical properties of AgNP mineralization peptides were different from those of AuNP mineralization peptides (Figure S1). A tryptophan residue was clearly an important amino acid for AuNP mineralization, and the pI and hydropathy values did not appear to coincide with the Au mineralization activity. A detailed investigation based on these differences would contribute to the elucidation of metallic nanoparticle mineralization peptides and their sequence design to control particle properties. 

In the image of the peptide array after AgNP mineralization activity evaluation, peptides demonstrating strong Ag mineralization are signified by a low summed color intensity value. The TOP10 peptides (re-named AgMP1–10; Ag mineralization peptide 1–10) and the WORST10 are listed with their physicochemical properties in Table 1 and Table 2, respectively. Interestingly, nine peptides of the TOP10 peptide sequences were found to have a unique motif, specifically EE and E[X]E (E: glutamic acid, and X: any amino acid), whereas none of these motifs were found in the WORST10 peptides (Table 1 and Table 2). The motif collectively possessing glutamic acids would be an important factor for AgNP mineralization by peptides.

### 2.2. Ag Nanoparticle Synthesis by Screened Mineralization Peptides 

**Figure 2:**
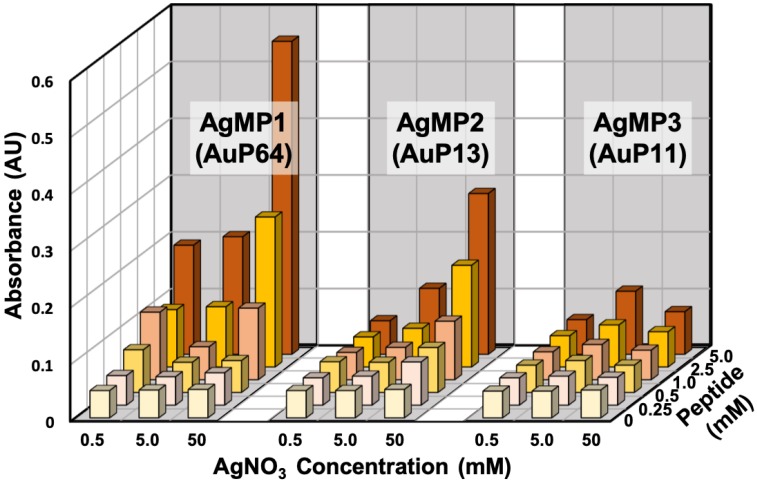
Silver nanoparticle (AgNP) synthesis using the top three (TOP3) screened peptides (AgMP1, AgMP2, and AgMP3) from the peptide array. Mineralization was evaluated based on absorbance intensity (450 nm) in the presence of different concentrations of peptides (0, 0.25, 0.5, 1.0, 2.5, and 5.0 mM) and AgNO_3_ (0.5, 5.0, and 50 mM).

The AgNP mineralization activity of the top three (TOP3) peptides (AgMP1; AESEHEWEVA, AgMP2; EEPHWEEMAA, and AgMP3; PEESQEGWMA) was further investigated for use in the one-pot green synthesis of AgNPs in aqueous solution. Herein, in the presence of different peptide and AgNO_3_ concentrations, AgNP mineralization was demonstrated (Figure 2). Particle synthesis by AgMP1 and AgMP2 was confirmed easily by the naked eye, and orange/yellow pigments derived from synthesized AgNPs were observed, whereas no color change was found in the presence of AgMP3 or without peptide. Therefore, it was shown that for the identified peptides, at least two can function in AgNP mineralization. In addition, according to the increase in peptide and AgNO_3_ concentrations, the increase in absorbance signals at 450 nm, indicative of AgNP mineralization, was found in the presence of AgMP1 and AgMP2, whereas no significant change was found in the presence of AgMP3 and in the absence of peptide. When the reaction was conducted with 5 mM of each peptide (AgMP1 and AgMP2) and 50 mM AgNO_3_, maximum absorbance signals were observed. 

**Figure 3:**
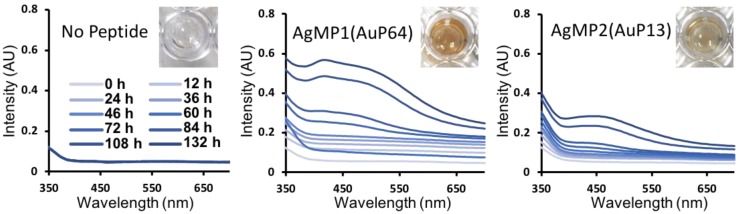
Time-dependent changes in absorption spectra of silver nanoparticles (AgNPs) synthesized by screened peptides, including AgMP1(AuP64) and AgMP2(AuP13). The mineralization was monitored until 132 h in the presence of each mineralization peptide (5 mM) and AgNO_3_ (50 mM). Images of solutions containing AgNPs synthesized by each peptide at 132 h are included.

To further characterize the mineralization mediated by peptides, time-course analyses of AgNP synthesis were performed, showing gradual AgNP synthesis (Figure 3). Clearly, different absorbance spectra were obtained by using AgMP1 and AgMP2, whereas no significant absorbance was found in the negative control (without peptide). The solution containing AgNPs synthesized by AgMP1 had two absorbance peaks at 420 nm and one broad peak at approximately 490 nm. The AgMP2-based solution revealed one broad absorbance peak at 450 nm. These spectra are expected to be derived from localized surface plasmon resonance (LSPR) by synthesized AgNPs. As the LSPR wavelength depends on the shape, size, and agglomeration state [25,26,27], it was suggested that the synthesized AgNPs have different characteristics. 

### 2.3. Transmission Electron Microscopic (TEM) Observation of AgNPs Synthesized by Screened Mineralization Peptides 

A TEM observation of synthesized AgNPs by each peptide (AgMP1 and AgMP2) was conducted (Figure S2). These two solutions contained two types of particles, large agglomerate and small spherical particles. Interestingly, the morphologies of agglomerate particles were obviously different in each sample. In the presence of AgMP1, large agglomerates made by vertically overlapping nanoplates (mainly triangle nanoplates) with sizes of 250 nm to 1 μm were found. To the best of our knowledge, there are no reports of a silver agglomerate with this unique morphology. In general, the triangle silver nanoplate revealed absorbance at 420 nm and the red-shifted region [27,28]. Therefore, two absorbance peaks at 420 and 490 nm might be observed in the solution containing AgNPs synthesized by AgMP1. In addition, as an increased absorbance in the whole range of wavelengths was found, the aggregation of biomineralized AgNPs seems to have occurred. This was supported by TEM observation; large agglomerates were found in the solution. From the size evaluation of more than 350 randomly selected AgNPs, the size of small spherical nanoparticles was 19.1 ± 9.3 nm. The AgNPs synthesized by AgMP2 showed large agglomerates with a coral morphology and a smaller size range than that of AgNPs derived from AgMP1 (200 to 750 nm). The small spherical nanoparticles were also smaller (4.9 ± 2.4 nm) than the AgNPs synthesized by AgMP1. 

## 3. Discussion

Using a list of AuNP-binding peptides synthesized on a peptide array, AgNP mineralization activity was evaluated. As a result, more than 50 AgNP mineralization peptides were effectively isolated (Figure 1). This is probably because Au and Ag have similar metallic properties and the utilized peptide library contains a large number of tryptophan molecules, which could play an important role as electron donors for particle formation. The result obtained here suggests that the 200 peptides have potential for the synthesis of other nanoparticles. However, the AgNP mineralization activity of isolated peptides was not consistent with that of AuNPs. This was very interesting because this observation reveals the potential of peptide isolation for metallic species-specific mineralization, through further comparative studies with other metals. 

From the sequence analysis of isolated AgNP mineralization peptides, EE and E[X]E (E: glutamic acid, and X: any amino acid) motifs were found. For this kind of analysis, it is beneficial to use peptide array technology, because the activity information could be obtained from all peptides, whereas it is difficult to use a positive screening technique including phage display library-based screening. In addition, the results indicated that the AgMP1 and AgMP2 peptides have two and three EE and E[X]E (E: glutamic acid, and X: any amino acid) motifs in their sequence, respectively, whereas AgMP3 has only one. This observation suggested that the number of EE and E[X]E (E: glutamic acid, and X: any amino acid) motifs is important for peptide-based AgNP mineralization in solution.

As shown in Figure 3, the AgNP mineralization reaction was still occurring, even after 132 h. In the case of AuNP mineralization, when G1 (ETGHHIWEWM) and B3 (ASHQWAWKWE) peptides were used, AuNPs of 2.6 ± 1.2 nm and 34.7 ± 6.7 nm were synthesized, respectively [15]. Interestingly, the reaction for smaller particle synthesis with an irregular shape reached a plateau within 10 min, whereas the synthesis of larger particles with unique morphologies, including triangle nanoplate and decahedron particles, required approximately 12 h [15]. As AgNPs with unique morphologies were also observed after long reaction times in this study, the difference in reaction kinetics seems to be a key factor for the formation of different sizes and morphologies of AgNPs, as discussed in a previous study on AuNP synthesis regulation [29,30,31]. In addition, further investigation of the interaction between peptides and specific crystal facets should be performed to elucidate the morphological regulation, because various biological molecules bound to a specific crystal surface are suggested to contribute to morphological regulation in the biomineralization process [32,33,34,35,36].

In conclusion, using a peptide array comprising an AuNP-binding peptide library, AgNP mineralization peptide screening was performed and identified approximately 50 different peptide candidates. From these sequence analyses, some unique characteristics, including amino acid frequency and physicochemical properties with a unique sequence motif, specifically EE and E[X]E (E: glutamic acid, and X: any amino acid), were found. Interestingly, among the TOP3 AgNP mineralization peptides isolated from the peptide array, two peptides (named AgMP1 and AgMP2) were revealed to possess AgNP-synthesis activity resulting in different morphologies. Moreover, it should be noted that although detailed analyses were only performed for two peptides screened in this study, the investigation could be expanded to the entire array of peptides. A comprehensive analysis using various peptides might contribute to the elucidation of AgNP mineralization by peptides and peptide design for morphologically controlled AgNP synthesis through a one-pot green course. The investigation of particle properties of each AgNP synthesized by the peptides might also lead to particle applications in various fields.

## 4. Materials and Methods 

### 4.1. Peptide Array Synthesis Using Spot Technology 

We used a cellulose membrane (grade 542; Whatman, Maidstone, UK), activated by β-alanine as the N-terminal basal spacer, as reported previously [37,38]. With a peptide auto-spotter (ResPepSL; Intavis AG, Köln, Germany), each activated Fmoc amino acid (0.5 M) was spotted on the membrane, as per the manufacturer’s instructions. After adding the first amino acid residue, the remaining amino groups were blocked with 4% acetic anhydride. The membrane, deprotected with 20% piperidine in N,N′-dimethylformamide (DMF), was subsequently washed thoroughly with DMF, followed by ethanol at each amino acid elongation process. After the final deprotection step, side-chain-protecting groups were deprotected using a solution of m-cresol:thioanisol:ethandithiol:trifluoroacetic acid (1:6:3:40) for 3 h. The membranes were finally carefully washed with diethyl ether and ethanol and dried. 

### 4.2. Screening of AgNP Mineralization Peptides Using Peptide Array

To screen AgNP mineralization peptides, 200 AuNP-binding and/or mineralization peptides were synthesized on a cellulose membrane. The synthesized membrane was incubated in an aqueous solution of 500 mM AgNO_3_ in MilliQ water under fluorescent light. After incubation for 7 h, individual peptide array spots were observed to change color, indicative of AgNP mineralization. After washing twice with MilliQ water, the mineralization activity of each peptide was evaluated quantitatively, based on the color intensity derived from AgNP mineralization activity onto peptide spots. This was measured from digitized images of the peptide array after the mineralization experiment (ImageQuant TL software, GE Healthcare, Tokyo, Japan). 

### 4.3. AgNP Synthesis and Characterization Using Screened Mineralization Peptides 

To evaluate the effects of AgNP mineralization peptides on AgNP synthesis in free aqueous solution (not on the peptide array), chemically synthesized and purified peptides were added to the reaction solution. The peptide was synthesized following the standard Fmoc-based solid-phase protocol, with a Respep SL automatic peptide synthesizer (Intavis AG, Köln, Germany). Briefly, Fmoc protected amino acid residues were applied to the TentaGel Resin stepwise for elongation of the peptide chain. The synthesized peptide was deprotected with 20% piperidine in DMF and cleaved from the scaffold resin by the cleavage cocktail containing TFA, water, thioanisole, phenol, EDT, and TIPS (82.5:5:5:2.5:1). Peptides were precipitated in cold diethyl ether and dissolved in 30% acetonitrile for storage in a form of freeze-dried powder. Purification was performed with an ODS-80TS column (Tosoh Corp., Tokyo, Japan) and a high-performance liquid chromatography (HPLC) system (LC-20AR, CBM-20A, SIL-20AC, CTO-20AC, SPD-20AV, Shimadzu Corp., Kyoto, Japan), before measuring the molecular weight by matrix assisted laser desorption/ionization mass spectrometry (AXIMA-CFRPlus, Shimadzu Corp.) (Figure S3). The final purity of the peptide was confirmed to be > 85% using an ODS-100Z column (Tosoh Corp.) and the HPLC system (Shimadzu Corp.)

Three different peptides comprising three AgMPs screened using a peptide array (AgMP1; AESEHEWEVA, AgMP2; EEPHWEEMAA, and AgMP3; PEESQEGWMA) were investigated. The peptide powders were dissolved in 100% DMSO (100 mM peptide). After confirming that DMSO did not affect Ag mineralization, different volumes of the peptide solution were added to AgNO_3_ containing MilliQ water for Ag mineralization evaluation.

TEM analysis was performed using a Hitachi H7650 microscope (Hitachi, Tokyo, Japan), operating at a working voltage of 100 kV. Specimens were prepared through the drop-casting of 1.5 µL of the sample dispersion onto a formvar-coated 200-mesh Cu grid (Nisshin EM, Tokyo, Japan) and washed with MilliQ water twice. The average sizes (± SD) of nanoparticles were obtained by manually counting >350 randomly selected particles in TEM images. UV-vis optical absorbance of the AgNPs was delineated using a microtiter plate reader (PowerScan 4, DS Pharma Biomedical Co., Ltd., Osaka, Japan).

## Figures and Tables

**Table 1:** List of Ag mineralization peptides (TOP10) screened, and their physical properties.

Peptide No.	Sequence	Mineralization Activity ^1^	pI ^2^	GRAVY2 ^2^
AuP64 (AgMP1)	AESEHEWEVA	162.9	4.09	−1.11
AuP13 (AgMP2)	EEPHWEEMAA	168.2	4.09	−1.42
AuP11 (AgMP3)	PEESQEGWMA	168.2	3.67	−1.4
AuP112 (AgMP4)	NWELEEHSAS	169.4	4.24	−1.41
AuP139 (AgMP5)	ETEWLGHETL	174.7	4.24	−0.88
AuP41 (AgMP6)	WSEETEMWPL	177.8	3.67	−0.97
AuP82 (AgMP7)	WQENSMEENW	183.9	3.67	−2.17
AuP180 (AgMP8)	HWWWEHEMEH	185.1	5.22	−2.09
AuP165 (AgMP9)	EGSDHPSWNQ	186.0	4.35	−2.17
AuP28 (AgMP10)	PEEGPHSLWH	186.3	5.23	−1.49

^1^ Using Image quant software, the mineralization activity of each peptide was determined through the quantitative analysis of spot color intensity for peptide array images. Average values for peptide spots are shown from triplicate independent experiments. ^2^ Based on the ProtParam tool in ExPASy (http://web.expasy.org/protparam/), the isoelectric point (pI) and the grand average of the hydropathy value (GRAVY) were shown.

**Table 2:** List of Ag mineralization peptides (WORST10) screened and their physical properties.

Peptide No.	Sequence	Mineralization Activity ^1^	pI ^2^	GRAVY ^2^
AuP77	YWASHKHWWW	221.2	8.61	−1.42
AuP173	WMMWGWVHEI	221.6	5.24	0.27
AuP65	TQWHEWHWYQ	221.9	5.98	−2.16
AuP155	NWTHWSTTQH	222.0	6.92	−1.81
AuP137	VHYGSQIEWG	222.7	5.24	−0.53
AuP95	AHALWIWHKT	223.0	8.76	−0.09
AuP125	TTWHGFPWAG	223.1	6.74	−0.42
AuP74	VLWRHEWAWK	223.1	8.75	−0.80
AuP116	WHHWAQGWHG	223.5	7.02	−1.48
AuP117	YEAVSTTWQS	224.0	4.00	−0.62

^1^ Using Image quant software, the mineralization activity of each peptide was determined through the quantitative analysis of spot color intensity for peptide array images. Average values for peptide spots are shown from triplicate independent experiments. ^2^ Based on the ProtParam tool in ExPASy (http://web.expasy.org/protparam/), the isoelectric point (pI) and the grand average of the hydropathy value (GRAVY) are shown.

## Supplementary Materials

Supplementary materials can be found at https://www.mdpi.com/1422-0067/21/7/2377/s1.

## Abbreviations

AgNP	Silver nanoparticle
AgMP	Silver mineralization peptide
AuNP	Gold nanoparticle
DMF	N,N′-dimethylformamide
pI	Isoelectric point
