# Supplementary material for: Array-Based Screening of Silver Nanoparticle Mineralization Peptides
Source: Int J Mol Sci. 2020 Mar 30;21(7):2377. doi: 10.3390/ijms21072377 (PMC7178033; doi:10.3390/ijms21072377)
Supplement: Supplementary file 1 [file ijms-21-02377-s001.pdf]

International Journal of
Molecular Sciences

MDPI

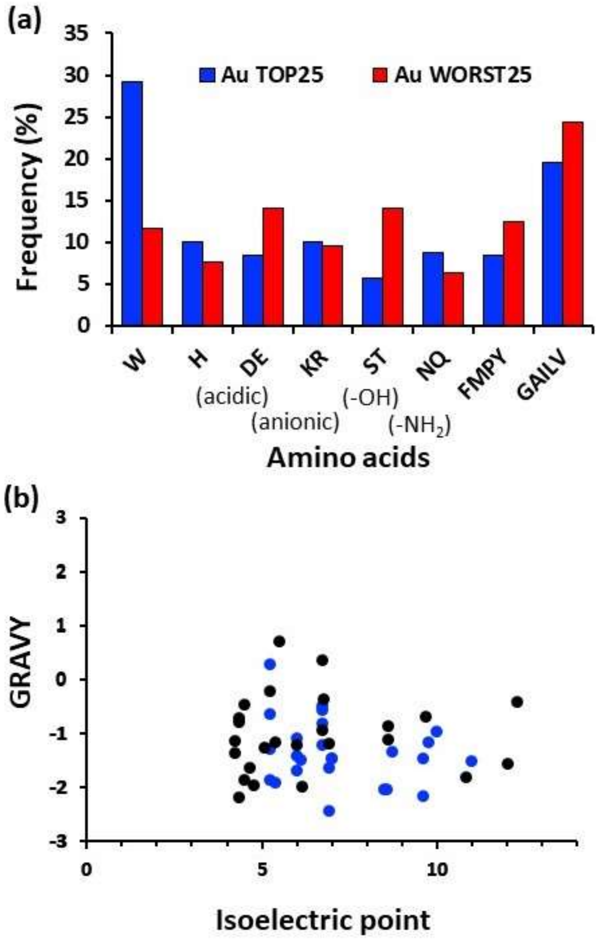

Figure S1. Characterization of previously reported AuNP mineralization peptides. (a) Amino acid frequencies in high-mineralization peptides (TOP25) and low-mineralization peptides (WORST25) from the brightness evaluation from each peptide spot using ImageJ. (b) Physicochemical properties of high and low mineralization peptides. Physicochemical properties of high binding TOP25 peptides (Blue circle) and worse binding WORST25 peptides (Black circle) based on values of pI and GRAVY (the grand average of hydropathy). The GRAVY value is defined as the average hydropathy value for all amino acids; therefore, a high GRAVY value indicates hydrophobicity.

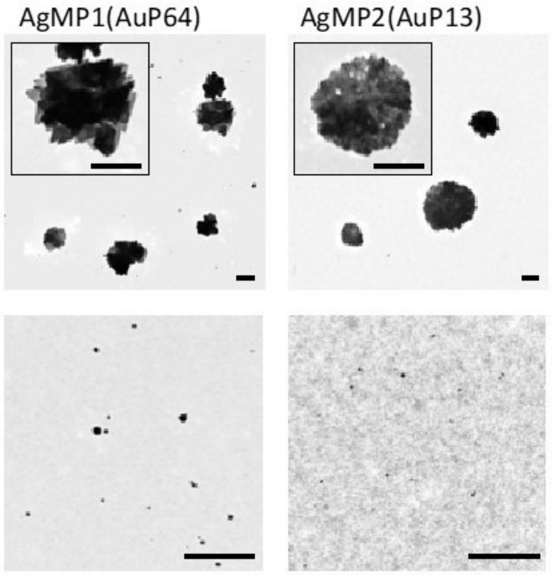

Figure S2. Transmission electron microscopic observation of silver nanoparticles (AgNPs) synthesized by two screened peptides (AgMP1 and AgMP2). The samples were prepared in the presence of peptides (5 mM) and $AgNO_3$ (50 mM). Scale bar indicates 250 nm.

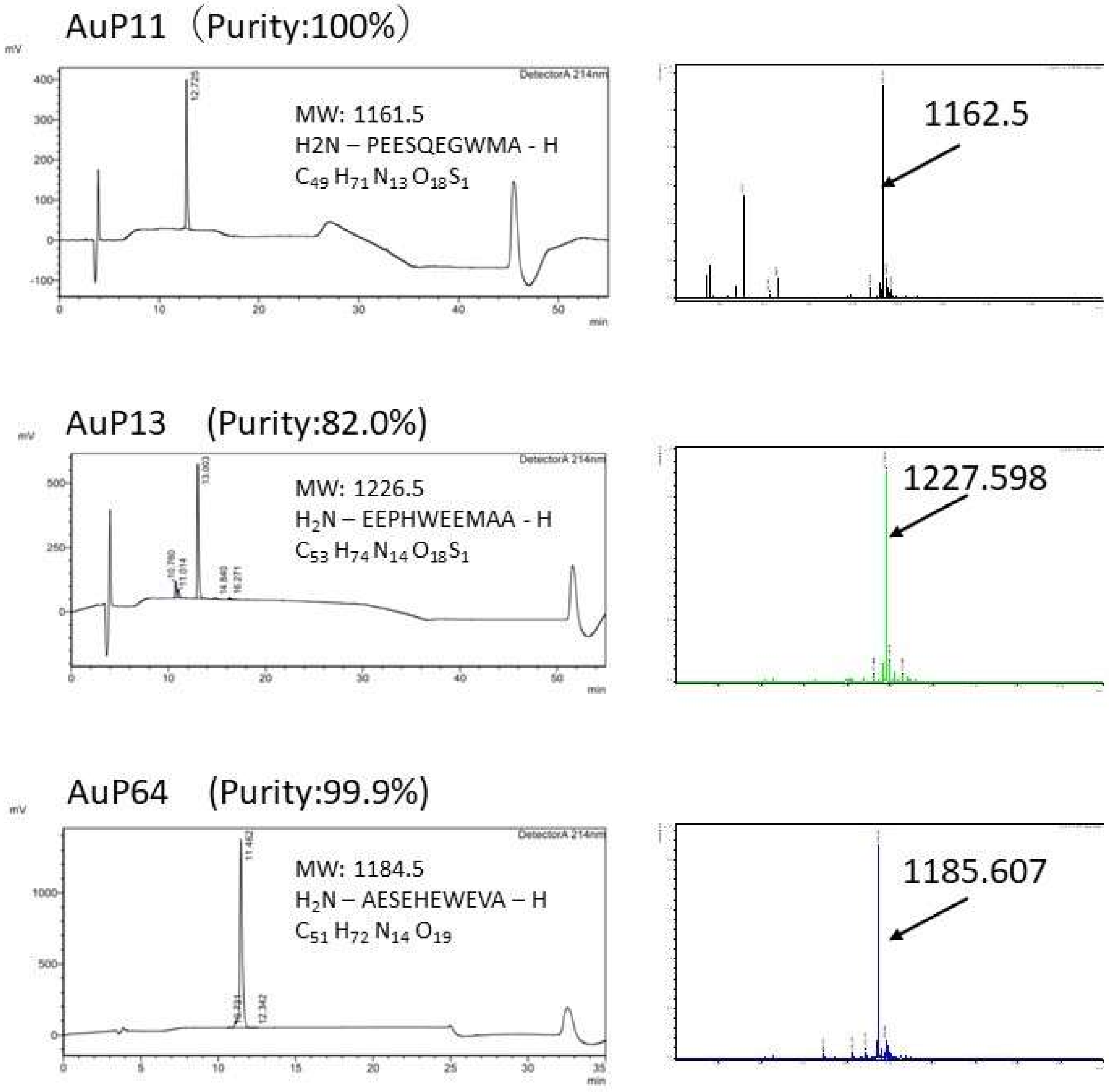

Figure S3. Confirmation of peptide purity by HPLC and MS.

© 2020 by the authors. Licensee MDPI, Basel, Switzerland. This article is an open access article distributed under the terms and conditions of the Creative Commons Attribution (CC BY) license (http://creativecommons.org/licenses/by/4.0/).
